# Clinical Advances in Viral-Vectored Influenza Vaccines

**DOI:** 10.3390/vaccines6020029

**Published:** 2018-05-24

**Authors:** Sarah Sebastian, Teresa Lambe

**Affiliations:** The Jenner Institute, University of Oxford, Old Road Campus Research Building, Headington, Oxford OX3 DQ, UK; sarah.sebastian@ndm.ox.ac.uk

**Keywords:** viral vectors, influenza, clinical trials

## Abstract

Influenza-virus-mediated disease can be associated with high levels of morbidity and mortality, particularly in younger children and older adults. Vaccination is the primary intervention used to curb influenza virus infection, and the WHO recommends immunization for at-risk individuals to mitigate disease. Unfortunately, influenza vaccine composition needs to be updated annually due to antigenic shift and drift in the viral immunogen hemagglutinin (HA). There are a number of alternate vaccination strategies in current development which may circumvent the need for annual re-vaccination, including new platform technologies such as viral-vectored vaccines. We discuss the different vectored vaccines that have been or are currently in clinical trials, with a forward-looking focus on immunogens that may be protective against seasonal and pandemic influenza infection, in the context of viral-vectored vaccines. We also discuss future perspectives and limitations in the field that will need to be addressed before new vaccines can significantly impact disease levels.

## 1. Introduction

Influenza virus is a respiratory pathogen that causes annual influenza epidemics affecting an estimated 15% of the global population with up to 645,000 deaths annually [1,2]. In addition, pandemic variants of the Influenza A virus (IAV) have been associated with upwards of 50 million deaths worldwide [3]. Vaccines are the primary intervention used to control seasonal influenza virus infection. Most licensed influenza vaccines are inactivated influenza vaccines (IIV) or live-attenuated influenza vaccines (LAIV) [4]. Inactivated influenza vaccines are commonly produced by growing the parent viruses in embryonated chicken eggs, or more recently, on cell lines, followed by a process of inactivation and dose standardization, typically performed on HA content (e.g., 15 µg/dose).

The WHO provides annual recommendations for the composition of influenza virus vaccines, approximately 6–9 months prior to upcoming influenza seasons in both the northern and southern hemispheres. Mismatch between the predicted strains and the actual circulating seasonal influenza variants occurs as frequently as every 3–4 years [5,6]. The development and implementation of vaccination regimens using a quadrivalent vaccine (that includes H3N2, H1N1, as well as both influenza B virus lineages, Yamagata and Victoria) has circumvented some of these limitations. Worryingly, however, vaccine effectiveness can be drastically affected even when seasonal vaccines are matched to circulating strains, as in the 2016/2017 season, when the HA in the vaccine was not post-translationally modified in the same manner as in the circulating influenza strains [7].

## 2. Major Vaccine Targets

### 2.1. Surface Antigens

IAV can be classified into subtypes based on the combination of surface-localized glycoproteins, hemagglutinin (HA-18 subtypes) and neuraminidase (NA-11 subtypes). Only H1N1, H3N2, and infrequently, H1N2 subtypes circulate regularly amongst humans while H2N2 has previously circulated. Of these, A/H1N1 and A/H3N2 subtypes consistently cause seasonal influenza epidemics.

Cellular entry of the influenza virus requires HA binding to sialic acid present on cell surface proteins. This binding is mediated by the receptor binding site (RBS) of HA which resides at the membrane-distal tip (globular head) of each monomer of the HA trimer [8]. Although the globular head domain of HA is more diverse than the membrane-proximal HA stalk, the RBS is relatively well-conserved required to preserve receptor-binding functionality. Currently licensed influenza vaccines principally act by inducing antibodies reactive to the viral surface proteins, predominantly HA. However, due to the high sequence variability in this protein, antibodies against one influenza virus can confer limited or no protection across types (or even within subtypes), and in some cases appear to be detrimental [9]. This is a considerable disadvantage, as IAV acquires mutations in the HA surface protein with comparative ease and relative frequency, thus generating antibody-escape variants. These ‘drift’ mutations are largely within the globular head domain of HA [10]. Alternatively, IAV can undergo more radical changes, acquiring HA or NA with an avian or porcine origin to form a distinct HA or NA subtype. Internal influenza antigens may also be replaced by this type of antigenic shift. The former drift process can cause seasonal epidemics, while the latter can generate novel subtypes with pandemic potential [11].

There is therefore an obvious and continuing need to improve seasonal vaccines or develop a universal vaccine, containing an antigenic target conserved in all influenza strains. A number of vaccination strategies have progressed to advanced preclinical and/or clinical testing (comprehensively reviewed in [12]). One obvious limitation of the currently licensed influenza vaccines is the narrow focus of responses toward distinct influenza viruses. Sought-after vaccine characteristics in the influenza field are the ability to generate broader responses, against viral variants other than just the vaccine-encoded ones, as well as the ability to generate robust long-lived immunity.

### 2.2. Internal (Non-Surface) Antigens

There are a number of internal influenza virus antigens that are readily recognized by the cellular immune response, including nucleoprotein (NP), polymerase basic 1 (PB1), and matrix 1 (M1) proteins. Cytotoxic T lymphocyte (CTL) responses do not inhibit viral entry or replication but can curb viral spread and limit transmission. IAV challenge work performed in the 1980s demonstrated that volunteers with measurable MHC class I-restricted CTLs prior to infection cleared the virus more efficiently [13]. More recent challenge studies have identified that higher frequencies of peripherally circulating IFN-γ-secreting CD4^+^ T cells that recognize peptides from NP and M1 correlated with less viral shedding and reduced symptom severity [14]. In addition, work investigating the cellular immune response during the 2009 pandemic identified that individuals with higher frequencies of pre-existing cross-reactive CD8^+^IFN-γ^+^ T-cells recognizing epitopes in PB1, M1, and NP had milder symptoms and lower risk of viral shedding following pandemic influenza infection [15]. These findings were subsequently corroborated by a larger cross-sectional observational community study, which found that NP-directed IFN-γ-secreting T-cells were associated with a decreased risk of viral shedding following influenza infection [16]. Of importance, T-cell responses to NP (≥20 SFU/10^6^ PBMC) were present in 43% of participants and correlated with a reduced risk of shedding virus by approximately two-thirds [16]. Describing and delineating this threshold level of cellular responses has clear implications for vaccine development and sets a realistic and achievable goal for vaccine manufacturers.

Building on this body of work, and essential to IAV vaccine development, is the identification of antigenic targets that are immunogenic and warrant inclusion in putative vaccine technologies. Importantly, both CD8^+^ and CD4^+^ T cells have demonstrated cross-reactivity to IAV strains to which individuals have not previously been exposed to, e.g., H1N1, H3N2, H2N2, and avian strains H5N1 and H7N9 viruses [17,18,19,20,21]. The major antigenic targets of heterosubtypic T-cells are epitopes in the highly conserved internal proteins of influenza, namely PB1, M1, and NP. The immunodominance of these proteins has been observed in a number of studies [15,20]; in addition to the high proportion of responders, the frequencies of T-cell responses are also highest to these antigens [20]. Thus, cytotoxic lymphocytes against conserved influenza antigens have the potential to recognize a range of virus strains, including those with pandemic potential. The challenge for the vaccinology field is to translate this knowledge into immunogenic, efficacious, and safe vaccines.

## 3. Viral Vectors as a Platform Technology

Viral vectors are an attractive vaccine modality, as they are able to efficiently transduce cells at the immunization site, resulting in de novo synthesis of the immunogen. The antigen is expressed at high levels and in its native conformation, characteristics which give rise to strong humoral and cellular immune responses. At the same time, the viral vector itself acts as an adjuvant, stimulating the innate immune system, which in turn enhances the adaptive, antigen-specific response. Viral vectors can be fully replication-competent, attenuated, or replication-deficient, with the latter possessing the best safety profiles. An advantage of replicating vectors is the amplification of antigen expression in each replication cycle, although the transgene must then compete with equally-amplified vector antigens for immunogenicity. Viral vectors have been shown to be efficacious after a single dose, and can induce long-lived immunity [22]. In addition, repeat immunization using viral vectors is possible without a negative impact on antigen immunogenicity [23]. Another advantage of the viral vector platform over current seasonal influenza vaccines are egg-independent production methods: viral vectors can be manufactured to high yields in bioreactors using well-characterized cell lines. To date, several viral-vectored vaccines have been licensed for use in humans; these are based on an attenuated yellow fever virus vector (Dengvaxia against Dengue fever and IMOJEV against Japanese encephalitis [24,25]), replication-deficient human adenovirus 5 vectors (Ad5-EBOV alone [26], and as part of GamEvac [27] for use in Ebola outbreaks), and an attenuated vesicular stomatitis virus vector (VSV-EBOV, as part of GamEvac for use in Ebola outbreaks [27]).

### 3.1. Viral-Vectored Influenza Vaccines in Clinical Trials

Out of a large range of preclinically tested vectored vaccines against influenza (comprehensively reviewed here [28]), three different types of viral vectors have progressed into clinical trials to date: the alphavirus VEEV, the poxvirus MVA, and several adenovirus species (Table 1 and Table 2). The majority of these constructs encode the HA protein (of either H1 or H5 IAV strains), and are designed to predominantly elicit an antibody-mediated immune response. In some cases, these humoral responses are broader and/or stronger than those achieved with seasonal influenza vaccines. As such, these vaccine candidates could replace current seasonal vaccines or could be used in a (pre-) pandemic setting of a known pandemic strain. In addition, several clinical trials have been performed with viral vectors encoding conserved viral antigens, which have the potential to induce cross-strain immune responses.

### 3.2. Alphavirus Vector

Alphaviruses are small single-stranded RNA viruses, many of which are transmitted by mosquitoes. Several alphavirus species have been explored preclinically as replication-deficient vaccine vectors against an array of pathogens and cancer [29,30], but this research has only translated into few clinical trials. In two of these trials, performed 10 years ago, Venezuelan equine encephalitis virus (VEEV) was tested as a vaccine vector carrying the influenza H3 HA gene, in both young and older adults. Trial results have not been published in peer-reviewed format, but a summary is provided on the sponsor’s website [31]. As stated, 80% and 86% of vaccinees achieved HI titers ≥40 after one or two immunizations, respectively. A measurable T-cell response to the antigen remained significantly elevated over 4 months. A similar trial in adults over the age of 65 years was less immunogenic, with only 50% of vaccinees achieving a 4-fold increase in HI titer, even after the second dose of vaccine. The alphavirus platform, which was developed by AlphaVax, was subsequently acquired by Novartis and is now held by GSK. Plans for future studies using this platform are not known at present.

### 3.3. Adenoviral Vectors

Adenoviruses are non-enveloped icosahedral double-stranded DNA viruses which can be ‘vectorized’ and rendered replication-deficient by deletion of essential genes. The viral genome can be engineered to carry antigen cassettes of up to 7 kb in size; adenoviruses have been tested extensively as gene therapy and vaccine vectors in humans, with highly acceptable safety profiles [32]. The most frequently used adenoviral vector is based on human adenovirus 5 (Ad5), but high levels of pre-existing immunity against this common respiratory pathogen in humans has led to the more recent development of vectors from rare human adenovirus serotypes or indeed from simians.

### 3.4. Human Adenovirus 5 (Ad5)

Several different adenoviruses have been assessed as influenza vaccine vectors in humans. The first trial of a human Ad5-vectored vaccine carrying an H1 HA antigen explored non-invasive administration routes (topical and intranasal) to circumvent the need for medically trained personnel during vaccination campaigns [33]. While the epicutaneous administration route proved disappointing (minimal increase in HI titers at the highest dose even after a booster application), intranasal vaccination (with a relatively low dose of 5 × 10^8^ vp) did result in modest systemic immunogenicity (as measured by serum HI titers). Reassuringly, in this group, no negative correlation was seen between immune response to HA and pre-existing anti-vector titers, although group size was small (*n* = 6). Further dose-escalating trials with this intranasally-administered Ad5 vector, encoding HA from H1N1 or H5N1, are ongoing [34,35].

Another promising mucosal delivery route which has been explored using the replication-deficient Ad5 vector is the oral route. In these trials, the adenoviral genome encoded both influenza HA as well as a small dsRNA molecule which acts as an adjuvanting TLR3 ligand [36,37]. In an initial trial, the vaccine was administered in the form of an enteric-coated capsule which contained lyophilized vector. Disappointingly, no induction of HI titers was observed in the first study, although a measurable T-cell response against the HA antigen was seen (3-fold increase from pre-vaccination levels, to an average of 60 IFN-g SFC/10^6^ PBMC) [36]. In order to delineate which intestinal site best responds to immunization, the Ad5-vectored vaccine was directly delivered to either the jejunum or ileum via radio-controlled capsules [38]. In this study, the ileum was found to be more efficient in inducing an antibody-based immune response. These results were taken into account in a subsequent trial, where tablet formulation was optimized for vaccine release in the ileum, and the vaccine dose was increased by 10-fold (to 10^11^ iu). These improvements resulted in substantial neutralizing antibodies against HA 4 weeks post-immunization (4-fold rise in HI titers as well as microneutralization titers in 11 out of 12 vaccinees, no change in titers in placebo group) [37]. The study authors also examined durability of the immune response, and found that 75% of vaccines still had HI titers ≥40 after 6 months. As in earlier trials with intranasally administered Ad5, the oral vaccine was well-tolerated, and there was no impact of pre-existing immunity to the Ad5 vector on immunogenicity towards the encoded antigen. These encouraging results led to a recently completed Phase II study to test the efficacy of this orally delivered vaccine candidate in an influenza challenge. A total of 180 participants received either the Ad5-HA vector (oral), the currently licensed quadrivalent inactivated influenza vaccine QIV (intramuscular) or a placebo, and were challenged with a matched H1N1 influenza strain 3 months later. Due to the very recent completion of the study, the results have yet to be published in a peer-reviewed journal, but the sponsor has released an announcement that the vaccine resulted in a statistically significant reduction in influenza infection compared to placebo control (as measured by virus shedding), and that only 37% of vaccinees developed influenza compared to 44% of QIV recipients and 71% of the control group [39].

### 3.5. Human Adenovirus 4 (Ad4)

Oral delivery of an adenoviral influenza vaccine has also been tested by another group, with one major difference compared to the trials described above: instead of replication-deficient Ad5, replication-competent Ad4 was used as the vaccine vehicle. This replicating adenoviral backbone has been used successfully by the US military for oral vaccination against Ad4-mediated respiratory disease [40], and it was thought that it might therefore also serve as an effective vector to carry heterologous antigens. Unfortunately, in a dose-escalation trial (10^7^–10^11^ vp) with 166 participants, only between 4% and 19% of vaccinees seroconverted to H5 HA even after 3 doses, whereas Ad4 seroconversion rates were found to be between 30% and 90% after 3 doses [41]. This result suggests that the heterologous influenza antigen (H5 HA) may be poorly immunogenic and/or may have been outcompeted by endogenous adenoviral proteins during the antibody-mediated immune response, likely due to the replication-competent nature of the vector. Interestingly, the vaccine vector did induce a modest but statistically significant cellular immune response to HA in 70% of vaccinees at the highest dose. In an amendment to the trial, certain participants were then boosted with inactivated H5N1 virus vaccine after 3 doses of the rAd4 vector, which led to 89% seroconversion to the influenza antigen in the highest dose group as measured by HI titers ≥40. The authors conclude that oral Ad4-H5 may act as a good priming agent for poorly immunogenic inactivated influenza vaccines such as H5N1. Further trials with this replication-competent vector are ongoing, testing alternative administration routes (intranasal, tonsillar) [42,43].

### 3.6. Chimpanzee Adenovirus (ChAdOx1)

ChAdOx1 was developed as an alternative to human adenoviral vaccine vectors (removing the problem of pre-existing anti-vector immunity) and is based on chimpanzee adenovirus isolate Y25 [44]. The clinical-stage ChAdOx1 influenza vaccine vector encodes the internal influenza virus antigens nucleoprotein (NP) and matrix 1 protein (M1) and is designed to elicit a cellular immune response to these highly conserved antigens. The ChAdOx1 NP+M1 vector has been used in two Phase I trials to date, with good safety and T-cell immunogenicity profiles after intramuscular administration [45,46]. In the first (dose-finding) trial, encouraging increases in antigen-specific T-cell responses were observed, with acceptable safety profiles in groups receiving up to 2.5 × 10^10^ vp of the ChAdOx1 vector [45]. These observations informed the vaccine dose used in the second trial, where all volunteers received 2.5 × 10^10^ vp [46]. In both trials, the ChAdOx1 NP+M1 vector was also tested in prime-boost combinations with a Modified Vaccinia Ankara (MVA) vector carrying the same influenza antigens (NP+M1). Specifically, a ChAdOx1 prime followed by an MVA boost with an 8- or 52-week interval was compared to an MVA prime with ChAdOx1 boost, also with 8- or 52-week intervals, in order to identify an optimal vaccination schedule [46]. The authors showed that such a two-dose heterologous regimen, in either order and at both intervals tested, was highly immunogenic, with the MVA/ChAdOx1 regimen resulting in slightly better T-cell durability in the follow-up period (18 months post-prime). In a separate arm of the same study, older volunteers (>50 years) received either a single dose of ChAdOx1 or a ChAdOx1-prime MVA-boost, to assess immunogenicity in older adults compared to the 18–46 year olds. While a single dose of ChAdOx1 was not sufficient to achieve maintenance of effector T-cell levels above baseline levels, an MVA boost after 8 weeks did improve T-cell durability up to 8 months [46].

### 3.7. Modified Vaccinia Ankara (MVA)

MVA is an attenuated poxvirus, derived from chorioallantois vaccinia virus Ankara (CVA) by more than 570 passages in primary chicken embryo fibroblasts [47]. Although it can be grown in a limited number of cell lines, MVA does not replicate in humans, and therefore possesses an excellent safety profile, even in immunocompromised populations [48]. When used as a vaccine vector, MVA induces high humoral as well as cellular antigen-specific immune responses, and is especially suitable for boosting previously primed immune responses [49].

The MVA-NP+M1 vector described above was also evaluated on its own, in a series of trials performed by the Jenner Institute [50,51,52,53]. A route and dose-finding study revealed that intramuscular administration was better tolerated than intradermal delivery, and that a dose of 1.5 × 10^8^ pfu was optimal with regard to reactogenicity and immunogenicity [50]. A single immunization with MVA-NP+M1 in younger adults (18–50 years) resulted in a robust antigen-specific T-cell peak one week after administration (median 1443 SFU/million PBMC) and maintenance of T-cell levels above pre-vaccination baseline up to 24 weeks at the highest dose [50]. In older adults (50–85 years), a similar T-cell response was seen, with a mean 8.5-fold increase compared to baseline, and durability of the response above baseline maintained to 12 weeks [53]. These encouraging results led to a Phase IIa efficacy study of MVA-NP+M1 in healthy adults, with 11 vaccinees and 11 control subjects challenged with H3N2 influenza virus 30 days post-immunization [52]. In this small study, the vaccine trended toward partial protection, as evidenced by only 2 vaccinees (compared to 5 controls) developing lab-confirmed influenza.

Previous pre-clinical work had demonstrated an adjuvanting effect of the MVA vector; specifically, when co-administered with TIV in mice, humoral immunity to HA (in the TIV) was boosted while cellular immunity against the MVA-encoded antigens (NP+M1) was maintained [54]. A clinical study was therefore conducted in which adults aged 50 and above were vaccinated with either the seasonal influenza vaccine plus a placebo, or in combination with MVA-NP+M1 [55]. The vaccine combination was safe and well-tolerated. The NP+M1-specific T-cell responses in vaccinees was significantly higher at peak compared to controls, as had been seen in previous studies with MVA-NP+M1 alone. The combination regimen also resulted in enhanced antibody-mediated immune responses against the surface hemagglutinin contained in the H3N2 component of the inactivated seasonal vaccine [55]. The MVA-NP+M1 vector has now progressed into an ongoing, large 2-year Phase IIb trial in older adults in Oxfordshire, UK, aiming to recruit over 2000 participants [56]. Volunteers receive the seasonal influenza vaccine in combination with either the MVA-NP+M1 vector or a saline placebo, given at the same time in the same arm, and are followed up for the duration of the UK influenza season. Primary outcomes of this trial are safety of the vaccine combination, and influenza-like illness, both the severity of symptoms and frequency thereof (self-reported), with a sub-cohort of 50 volunteers per group also being assessed on immunological endpoints. This trial is blinded and extends over a period of 2 years, and results are expected in late 2019.

Finally, it is worth mentioning that MVA-NP+M1 is not the only MVA vector that has been tested as an influenza vaccine candidate in the clinic. MVA encoding H5 HA has also been assessed in a trial, for induction of an antigen-specific antibody response and its potential use in (pre-)pandemic settings [57,58]. In this study, MVA-H5 or a control MVA were given at two different doses, either in a prime-only or a 4-week prime-boost regimen. A subset of volunteers was also given another booster immunization after one year. Two or more immunizations with MVA-H5 were necessary, as unlike MVA-NP+M1, which will boost a response in a human population previously exposed to any influenza A strain, there will only be a limited pre-existing humoral response to the H5 antigen in the general population. Therefore, a response against H5 HA first needs to be primed by the MVA-H5 vaccine itself. Immunizations were well-tolerated and resulted in an increase in average HI titers to >80 after 2 doses, at the higher dose (10^8^ pfu). This antibody response could be efficiently boosted by a further immunization after one year, after which average HI titers reached >640. Importantly, anti-H5 antibodies elicited by this vector were also able to neutralize an H5N1 virus from an antigenically distinct clade [57] as well as a newly emerged highly pathogenic avian H5N8 strain found in outbreaks on poultry farms [58].

## 4. Future Perspectives

There are a number of remaining hurdles on the road to generating, testing, and licensing improved viral-vectored influenza vaccines that may be effective in both pandemic and epidemic settings.

### 4.1. Improved Antigenic Targeting of HA

Structural and functional biology research have identified highly conserved and immunologically relevant antigenic sites on the HA immunogen. The current challenge is to convert these antigenic targets into tenable real-world vaccine solutions. Prominent vaccination strategies involve antibody-targeting to the conserved stalk of the HA protein or to conserved epitopes in the immune-dominant and diverse HA globular head domain.

### 4.2. The Receptor Binding Site (RBS) in the HA Globular Head Domain

Viral RBS are often poorly immunogenic, and broadly neutralizing antibodies targeting the RBS have been difficult to isolate, perhaps in part because of the relatively small footprint of the RBS. One of the first antibodies with heterosubtypic reactivity to be described, S139/1, a IgG2a that binds a conformational epitope adjacent to the receptor-binding site of the HA, is able to neutralize strains from multiple subtypes, e.g., H1, H2, H3, and H13 [59]. Crystal structure analysis of the S139/1 antigen-binding fragment (Fab) demonstrated that S139/1 is unusually dependent upon avidity for heterosubtypic neutralization [60,61]. This important insight not only highlights the RBS as an important heterosubtypic antigenic target but also details the type of humoral immune response that would be best targeted: bivalent antibody binding [60,61].

Other reports have described a number of broadly neutralizing antibodies that map to the apex of HA close to/or at the receptor-binding site; these RBS-binding antibodies have been isolated from vaccinees following seasonal influenza vaccination [62]. The isolation of single B-cells and subsequent sequencing of the heavy- and light-chain genes have led to the discovery of a “signature” which can aid in the identification of any potential RBS-directed antibodies after vaccination. Follow-on work has demonstrated that RBS-specific antibodies are far more common than originally thought and might realistically be induced following RBS-targeted vaccination [63,64]. Importantly, anti-RBS antibodies could have pandemic impact; a human monoclonal antibody, HNIgGA6, induced post natural-infection with H7N9, can neutralize the H7N9 virus both *in vitro* and *in vivo* by directly binding the receptor binding site [65].

### 4.3. The Conserved Stalk of the HA Protein

The most recent global IAV pandemic in 2009 was caused by an H1N1 variant and highlighted the importance of HA-specific anti-stalk antibodies [66,67]. Broadly neutralizing anti-stalk antibodies have been more readily identified after natural influenza infection, when compared to isolation post-seasonal influenza vaccination [67,68,69,70,71,72]. Of interest, IgA-mediated neutralization activity from human serum was found to be more effective than neutralization by IgG [73]. There are a number of vaccination approaches to induce anti-stalk antibodies that have been recently and comprehensively reviewed [74,75,76]. To summarize, the two principal approaches are (1) repeat vaccination with chimeric HA variants and (2) vaccination with HA that lacks the globular HA head domain. The former approach induces an immune response to the whole priming HA immunogen; subsequent repeat vaccinations with chimeras that share the stalk motif but carry a different head will boost the response against the stalk. This approach is now being assessed in a Phase I clinical trial [77]. The second approach involves vaccination with ‘headless constructs’, essentially stalk domains without the globular head. However, the head domain is believed to be crucial for HA stability. More recently, novel approaches have been adopted to better stabilize and thereby target the HA stalk; these include adding motifs to stabilize the headless HA, or using a ‘scaffold’ to prevent degradation [78,79,80].

### 4.4. Fundamental Understanding of Immunity

Natural influenza infection can induce long-lived and durable immunity; humoral immunity can be life-long, and influenza-specific T-cells have been shown to persist for 13 years [81]. However, it is unclear how long efficacious cellular immunity persists. While this scenario seems ideal to provide enduring protection, the impact of original antigenic priming and pre-existing memory responses on subsequent influenza vaccination remains to be resolved [74,82]. While current inactivated influenza vaccines are associated with short-lived immunity [83,84], ideal vaccines would limit terminal differentiation of immune memory cells. This would, upon re-exposure to variant influenza viruses, allow re-entry of memory B-cells into germinal centers to generate antigen-specific antibodies and generation of heterosubtypic effector T-cells from previously exposed memory T cells. Manipulation of immunological mechanisms with the aim of efficacious priming but not terminally differentiating represents the best strategy to protect vaccinees against variant influenza strains including both seasonal and pandemic strains.

In situ immune responses at the interface of pathogen and host tissue have been demonstrated to be critical in pre-clinical animal models of influenza infection [85,86,87]. However, by its nature, tissue-resident immunity in humans, and its role in disease, has been more difficult to characterize. For example, one important area to be explored is the role of mucosal versus serum antibodies in protection against influenza virus infection. Encouragingly, clinical data on this topic is accumulating [88,89,90], and this should impact on vaccine development programs.

### 4.5. Future Work Needed

In contrast to IAV, the animal reservoir for IBV is limited, thus facilitating the potential of vaccine eradication programs. Additionally, the severity of IBV disease in young children and older adults is similar to IAV-related disease. In spite of this, there is limited ongoing clinical trial research on innovative vaccines for this important respiratory pathogen. Whatever approach is taken, it is likely that vaccines that deliver multiple influenza-specific antigens (thus restraining viral escape mutants) will be best-placed to advance the influenza vaccine field for both IAV and IBV. There are a number of other influenza antigens that are current vaccine targets which might be considered in a combination approach.

### 4.6. Humoral Targets

The influenza ion channel M2 is expressed at high density on virus-infected cells, and its ectodomain is highly conserved across human IAVs. Vaccine approaches are being developed to induce antibodies against this conserved ectodomain.

The viral surface protein neuraminidase (NA) is also being explored as a vaccine target. Antibodies against NA interfere with virus release and can reduce the amount of influenza virus produced by infected cells. Recent studies have shown that animals immunized with adjuvanted NA can be protected against viral challenge [91] including prophylactic protection against avian H5N1 viruses and therapeutic protection from lethal influenza virus challenge [92]. Importantly, NA-reactive antibodies display broad heterosubtypic binding activity and are effectively induced to significant levels post-infection in humans [92].

Additional strategies to develop antibody-based HA-targeting vaccines include Computationally Optimized Broadly Reactive Antigen (COBRA) from consensus HA sequences.

### 4.7. Cellular Targets

A number of researchers are also investigating T-cell based influenza vaccines and are developing peptide or epitope-based approaches; several immune-dominant epitopes with limited variability in influenza antigens have been identified [93,94]. However, discrepancies exist between epitopes that are algorithmically predicted and those recognized in ‘real-world’ situations [95]. Additionally, while cellular immune responses can be induced against a large number of conserved epitopes, these are not necessarily protective, and currently there is a paucity of clinical studies identifying specific protective T-cell epitopes in humans. So far, studies have demonstrated that reduced disease severity is associated with higher frequencies of CD4^+^ T-cell epitopes spanning NP and M1 proteins. Some studies have suggested that exceedingly high antibody titres against a variant NP epitope, in a particular HLA-haplotype, may predispose toward autoimmune-mediated narcolepsy [96]. However, it is unlikely that a vaccine designed to predominantly induce cellular immunity will also induce sufficiently high humoral immunity to affect this pathway; additionally, NP-containing vaccines can be easily engineered to exclude this putative disease-causing epitope.

Regardless of the combination approach that may be taken, empirical-based design and iterative testing will be needed. Special attention should be paid to determination of dose and route of vaccination, to ensure that (1) the immune response is sufficient to clear the current infection, but also generates a memory response able to respond to a recurrent and varying pathogen and that (2) the immune response is best placed to clear a respiratory virus. 

## 5. Conclusions

Obvious limitations of current seasonal influenza vaccines and the continuing threat of future pandemics have made it clear that innovative vaccine solutions are needed. To address this need, influenza research over the past decade has focused on the discovery of new (universal) antigenic targets and improved vaccine modalities. Among the latter, those that have progressed into clinical trials are viral vectors, plasmid DNA ([97] and more), recombinant proteins [98,99], and polypeptide epitope sequences [100,101,102]. The focus of this review has been on viral vectors and their potential as strong candidates for protective influenza vaccines. While most viral-vectored vaccine candidates currently in the clinic are aimed at eliciting an antibody response against HA from specific clades, several other candidates were designed to induce T-cell responses against more conserved influenza proteins. An improved viral vector candidate could therefore contain a combination of antigenic targets, harnessing the potential of these vaccine vehicles to induce both cellular and humoral immune responses. Considering active, ongoing preclinical research in this area, the next five years carry the promise of exciting and impactful results.

## Figures and Tables

**Table 1:**
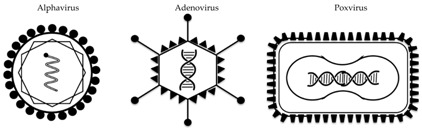
Characteristics of viral vectors used in clinical trials for influenza.

Vector	Alphavirus	Adenovirus	Poxvirus
**Example(s)**	VEEV	Ad5, rAd4, ChAdOx1	MVA
**Virus characteristics**	enveloped, ssRNA	non-enveloped, DNA	enveloped, DNA
**Transgene insertion capacity**	5 kb	7 kb	>20 kb
**Replication site**	cytoplasm	nucleus	cytoplasm
**Tropism**	lymphocytes, neuronal cells, fibroblasts	broad	broad
**Transgene-specific immune response**	Abs, T-cells	Abs, T-cells	Abs, T-cells
**Delivery method(s)**	intramuscular	intramuscular, intranasal, oral	intramuscular, intradermal, aerosol

**Table 2:** Viral-vectored influenza A vaccines in ongoing or completed clinical trials. VEEV: Venezuelan equine encephalitis virus. MVA: modified vaccinia virus Ankara. Ad: adenovirus.

Vector(s)	Antigen	Number of Participants (Age Range), Phase	Description of Trial	References	Clinical Trial Identifier, Sponsor
VEEV (alphavirus)	HA from H3N2	216 (18–40), I/II	Homologous prime-boost	Study completed, not published	NCT00440362 AlphaVax
		28 (>65), I/II	Homologous prime-boost	Study completed, not published	NCT00706732 AlphaVax
rAd4 (replication-competent human Ad)	HA from H5N1	166 (18–40), I	Oral route	[40]	NCT01006798 PaxVax
		51 (18–49), I	Intranasal, dose escalation		NCT01806909 PaxVax
		96 (18–49), I	Oral and tonsillar routes		NCT01443936 PaxVax
Ad5 (replication-deficient human Ad)	HA from H1N1 plus dsRNA adjuvant	180 (18–49), II	Oral route, with H1N1 challenge	Study recently completed	NCT02918006 VaxArt
		24 (18–49), I	Oral administration	[36]	NCT01688297 VaxArt
		8 (18–49), I	Oral, effect of tablet size and fasting		NCT03121339 VaxArt
		24 (18–49), I	Direct delivery to ileum by radio-controlled capsule	[37]	NCT01761123 VaxArt
	HA from H5N1 plus dsRNA adjuvant	54 (18–49), I	Oral administration	[35]	NCT01335347 VaxArt
	HA from H1N1	24 (20–31), I	Intranasal and epicutaneous	[32]	Vaxin
		60 (18–49), IIa	Intranasal dose-escalation (NasoVax)		NCT03232567 Altimmune
	HA from H5N1	48 (18–49), I	Intranasal dose-escalation		NCT00755703 Vaxin/Altimmune
ChAdOx1 (replication-deficient chimpanzee adenovirus) + MVA	NP+M1 from H3N2	15 (18–50), I	ChAdOx1 dose escalation, ChAdOx1 prime-MVA boost	[44]	NCT01623518 Jenner Institute
		48 (18–46) and 24 (>50), I	Heterologous prime-boost at different intervals	[45]	NCT01818362 Jenner Institute
MVA	HA from H5N1	80 (18–28), I/IIa	Immunogenicity at two doses; homologous prime-boost	[56,57]	NTR3401 Erasmus Medical Center
	NP+M1 from H3N2	28 (18–50) and 30 (50–85), I	Intradermal and intramuscular, adults and older adults	[49,52]	NCT00942071 Jenner Institute
		22 (28–45), IIa	Immunisation and H3N2 challenge	[50,51]	NCT00993083 Jenner Institute
		17 (>50), I	Co-admin with seasonal inactivated vaccine	[54]	NCT01465035 Jenner Institute
		6 (18–50), I	Bridging study: vector manufactured on new cell line	Study completed, not published	NCT03277456 Vaccitech
		2000+ (>65), IIb	Co-admin with seasonal vaccine; efficacy	Ongoing	NCT03300362 Vaccitech

## References

[1] Iuliano A.D., Roguski K.M., Chang H.H., Muscatello D.J., Palekar R., Tempia S., Cohen C., Gran J.M., Schanzer D., Cowling B.J. (2017). Estimates of global seasonal influenza-associated respiratory mortality: A modelling study. Lancet.

[2] Molinari N.A., Ortega-Sanchez I.R., Messonnier M.L., Thompson W.W., Wortley P.M., Weintraub E., Bridges C.B. (2007). The annual impact of seasonal influenza in the US: Measuring disease burden and costs. Vaccine.

[3] Johnson N.P., Mueller J. (2002). Updating the accounts: Global mortality of the 1918–1920 “Spanish” influenza pandemic. Bull. Hist. Med..

[4] Rajao D.S., Perez D.R. (2018). Universal Vaccines and Vaccine Platforms to Protect against Influenza Viruses in Humans and Agriculture. Front. Microbiol..

[5] Carrat F., Flahault A. (2007). Influenza vaccine: The challenge of antigenic drift. Vaccine.

[6] Monto A.S., Ansaldi F., Aspinall R., McElhaney J.E., Montano L.F., Nichol K.L., Puig-Barbera J., Schmitt J., Stephenson I. (2009). Influenza control in the 21st century: Optimizing protection of older adults. Vaccine.

[7] Zost S.J., Parkhouse K., Gumina M.E., Kim K., Diaz Perez S., Wilson P.C., Treanor J.J., Sant A.J., Cobey S., Hensley S.E. (2017). Contemporary H3N2 influenza viruses have a glycosylation site that alters binding of antibodies elicited by egg-adapted vaccine strains. Proc. Natl. Acad. Sci. USA.

[8] Bradley K.C., Galloway S.E., Lasanajak Y., Song X., Heimburg-Molinaro J., Yu H., Chen X., Talekar G.R., Smith D.F., Cummings R.D. (2011). Analysis of influenza virus hemagglutinin receptor binding mutants with limited receptor recognition properties and conditional replication characteristics. J. Virol..

[9] Kim J.H., Skountzou I., Compans R., Jacob J. (2009). Original Antigenic Sin Responses to Influenza Viruses. J. Immunol..

[10] Smith D.J., Lapedes A.S., de Jong J.C., Bestebroer T.M., Rimmelzwaan G.F., Osterhaus A.D., Fouchier R.A. (2004). Mapping the antigenic and genetic evolution of influenza virus. Science.

[11] Doherty P.C., Turner S.J., Webby R.G., Thomas P.G. (2006). Influenza and the challenge for immunology. Nat. Immunol..

[12] Berlanda Scorza F., Tsvetnitsky V., Donnelly J.J. (2016). Universal influenza vaccines: Shifting to better vaccines. Vaccine.

[13] McMichael A.J., Gotch F.M., Noble G.R., Beare P.A. (1983). Cytotoxic T-cell immunity to influenza. N. Engl. J. Med..

[14] Wilkinson T.M., Li C.K., Chui C.S., Huang A.K., Perkins M., Liebner J.C., Lambkin-Williams R., Gilbert A., Oxford J., Nicholas B. (2012). Preexisting influenza-specific CD4^+^ T cells correlate with disease protection against influenza challenge in humans. Nat. Med..

[15] Sridhar S., Begom S., Bermingham A., Hoschler K., Adamson W., Carman W., Bean T., Barclay W., Deeks J.J., Lalvani A. (2013). Cellular immune correlates of protection against symptomatic pandemic influenza. Nat. Med..

[16] Hayward A.C., Wang L., Goonetilleke N., Fragaszy E.B., Bermingham A., Copas A., Dukes O., Millett E.R., Nazareth I., Nguyen-Van-Tam J.S. (2015). Natural T Cell-mediated Protection against Seasonal and Pandemic Influenza. Results of the Flu Watch Cohort Study. Am. J. Respir. Crit. Care Med..

[17] Tu W., Mao H., Zheng J., Liu Y., Chiu S.S., Qin G., Chan P.L., Lam K.T., Guan J., Zhang L. (2010). Cytotoxic T lymphocytes established by seasonal human influenza cross-react against 2009 pandemic H1N1 influenza virus. J. Virol..

[18] Jameson J., Cruz J., Ennis F.A. (1998). Human cytotoxic T-lymphocyte repertoire to influenza A viruses. J. Virol..

[19] Agrati C., Castilletti C., Cimini E., Lapa D., Quartu S., Caglioti C., Lanini S., Cattoli G., Martini F., Ippolito G. (2014). Cellular and humoral cross-immunity against two H3N2v influenza strains in presumably unexposed healthy and HIV-infected subjects. PLoS ONE.

[20] Lee L.Y., Ha do L.A., Simmons C., de Jong M.D., Chau N.V., Schumacher R., Peng Y.C., McMichael A.J., Farrar J.J., Smith G.L. (2008). Memory T cells established by seasonal human influenza A infection cross-react with avian influenza A (H5N1) in healthy individuals. J. Clin. Investig..

[21] Quinones-Parra S., Grant E., Loh L., Nguyen T.H., Campbell K.A., Tong S.Y., Miller A., Doherty P.C., Vijaykrishna D., Rossjohn J. (2014). Preexisting CD8+ T-cell immunity to the H7N9 influenza A virus varies across ethnicities. Proc. Natl. Acad. Sci. USA.

[22] Ewer K.J., Lambe T., Rollier C.S., Spencer A.J., Hill A.V., Dorrell L. (2016). Viral vectors as vaccine platforms: from immunogenicity to impact. Curr. Opin. Immunol..

[23] Harrop R., Shingler W., Kelleher M., de Belin J., Treasure P. (2010). Cross-trial analysis of immunologic and clinical data resulting from phase I and II trials of MVA-5T4 (TroVax) in colorectal, renal, and prostate cancer patients. J. Immunother..

[24] Guy B., Noriega F., Ochiai R.L., L’Azou M., Delore V., Skipetrova A., Verdier F., Coudeville L., Savarino S., Jackson N. (2017). A recombinant live attenuated tetravalent vaccine for the prevention of dengue. Expert Rev. Vaccines.

[25] Appaiahgari M.B., Vrati S. (2010). IMOJEV((R)): A Yellow fever virus-based novel Japanese encephalitis vaccine. Expert Rev. Vaccines.

[26] Li J.X., Hou L.H., Meng F.Y., Wu S.P., Hu Y.M., Liang Q., Chu K., Zhang Z., Xu J.J., Tang R. (2017). Immunity duration of a recombinant adenovirus type-5 vector-based Ebola vaccine and a homologous prime-boost immunisation in healthy adults in China: Final report of a randomised, double-blind, placebo-controlled, phase 1 trial. Lancet Glob. Health.

[27] Dolzhikova I.V., Zubkova O.V., Tukhvatulin A.I., Dzharullaeva A.S., Tukhvatulina N.M., Shcheblyakov D.V., Shmarov M.M., Tokarskaya E.A., Simakova Y.V., Egorova D.A. (2017). Safety and immunogenicity of GamEvac-Combi, a heterologous VSV- and Ad5-vectored Ebola vaccine: An open phase I/II trial in healthy adults in Russia. Hum. Vaccines Immunother..

[28] De Vries R.D., Rimmelzwaan G.F. (2016). Viral vector-based influenza vaccines. Hum. Vaccines Immunother..

[29] Lundstrom K. (2014). Alphavirus-based vaccines. Viruses.

[30] Mogler M.A., Kamrud K.I. (2015). RNA-based viral vectors. Expert Rev. Vaccines.

[31] AlphaVax AlphaVax Clinical Experience. https://www.alphavax.com/clinical-experience.html.

[32] Humphreys I.R., Sebastian S. (2018). Novel viral vectors in infectious diseases. Immunology.

[33] Van Kampen K.R., Shi Z., Gao P., Zhang J., Foster K.W., Chen D.T., Marks D., Elmets C.A., Tang D.C. (2005). Safety and immunogenicity of adenovirus-vectored nasal and epicutaneous influenza vaccines in humans. Vaccine.

[34] Single-Ascending-Dose Study of the Safety and Immunogenicity of NasoVAX. https://ClinicalTrials.gov/show/NCT03232567.

[35] Safety and Immunogenicity Study of Adenovirus-Vectored, Intranasal Pandemic Influenza Vaccine. https://ClinicalTrials.gov/show/NCT00755703.

[36] Peters W., Brandl J.R., Lindbloom J.D., Martinez C.J., Scallan C.D., Trager G.R., Tingley D.W., Kabongo M.L., Tucker S.N. (2013). Oral administration of an adenovirus vector encoding both an avian influenza A hemagglutinin and a TLR3 ligand induces antigen specific granzyme B and IFN-gamma T cell responses in humans. Vaccine.

[37] Liebowitz D., Lindbloom J.D., Brandl J.R., Garg S.J., Tucker S.N. (2015). High titre neutralising antibodies to influenza after oral tablet immunisation: A phase 1, randomised, placebo-controlled trial. Lancet Infect. Dis..

[38] Kim L., Martinez C.J., Hodgson K.A., Trager G.R., Brandl J.R., Sandefer E.P., Doll W.J., Liebowitz D., Tucker S.N. (2016). Systemic and mucosal immune responses following oral adenoviral delivery of influenza vaccine to the human intestine by radio controlled capsule. Sci. Rep..

[39] Vaxart Vaxart News Releases. http://vaxart.com/NRfiles/VaxartAnnouncesReducedInfluenzaInPhase2InfluenzaChallenge013118.pdf.

[40] Radin J.M., Hawksworth A.W., Blair P.J., Faix D.J., Raman R., Russell K.L., Gray G.C. (2014). Dramatic decline of respiratory illness among US military recruits after the renewed use of adenovirus vaccines. Clin Infect. Dis..

[41] Gurwith M., Lock M., Taylor E.M., Ishioka G., Alexander J., Mayall T., Ervin J.E., Greenberg R.N., Strout C., Treanor J.J. (2013). Safety and immunogenicity of an oral, replicating adenovirus serotype 4 vector vaccine for H5N1 influenza: A randomised, double-blind, placebo-controlled, phase 1 study. Lancet Infect. Dis..

[42] Intranasal AD4-H5-VTN as an Adenovirus Vaccine. https://ClinicalTrials.gov/show/NCT01806909.

[43] Experimental AD4-H5-VTN Vaccine in Healthy Volunteers. https://ClinicalTrials.gov/show/NCT01443936.

[44] Dicks M.D., Spencer A.J., Edwards N.J., Wadell G., Bojang K., Gilbert S.C., Hill A.V., Cottingham M.G. (2012). A novel chimpanzee adenovirus vector with low human seroprevalence: Improved systems for vector derivation and comparative immunogenicity. PLoS ONE.

[45] Antrobus R.D., Coughlan L., Berthoud T.K., Dicks M.D., Hill A.V., Lambe T., Gilbert S.C. (2014). Clinical assessment of a novel recombinant simian adenovirus ChAdOx1 as a vectored vaccine expressing conserved Influenza A antigens. Mol. Ther..

[46] Coughlan L., Sridhar S., Payne R., Edmans M., Milicic A., Venkatraman N., Lugonja B., Clifton L., Qi C., Folegatti P.M. (2018). Heterologous Two-Dose Vaccination with Simian Adenovirus and Poxvirus Vectors Elicits Long-Lasting Cellular Immunity to Influenza Virus A in Healthy Adults. EBioMedicine.

[47] Mayr A., Hochstein-Mintzel V., Stickl H. (1975). Abstammung, Eigenschaften und Verwendung des attenuierten Vaccinia-Stammes MVA. Infection.

[48] Overton E.T., Stapleton J., Frank I., Hassler S., Goepfert P.A., Barker D., Wagner E., von Krempelhuber A., Virgin G., Meyer T.P. (2015). Safety and Immunogenicity of Modified Vaccinia Ankara-Bavarian Nordic Smallpox Vaccine in Vaccinia-Naive and Experienced Human Immunodeficiency Virus-Infected Individuals: An Open-Label, Controlled Clinical Phase II Trial. Open Forum Infect. Dis..

[49] Gilbert S.C. (2013). Clinical development of Modified Vaccinia virus Ankara vaccines. Vaccine.

[50] Berthoud T.K., Hamill M., Lillie P.J., Hwenda L., Collins K.A., Ewer K.J., Milicic A., Poyntz H.C., Lambe T., Fletcher H.A. (2011). Potent CD8+ T-cell immunogenicity in humans of a novel heterosubtypic influenza A vaccine, MVA-NP+M1. Clin Infect. Dis..

[51] Powell T.J., Peng Y., Berthoud T.K., Blais M.E., Lillie P.J., Hill A.V., Rowland-Jones S.L., McMichael A.J., Gilbert S.C., Dong T. (2013). Examination of influenza specific T cell responses after influenza virus challenge in individuals vaccinated with MVA-NP+M1 vaccine. PLoS ONE.

[52] Lillie P.J., Berthoud T.K., Powell T.J., Lambe T., Mullarkey C., Spencer A.J., Hamill M., Peng Y., Blais M.E., Duncan C.J. (2012). Preliminary assessment of the efficacy of a T-cell-based influenza vaccine, MVA-NP+M1, in humans. Clin. Infect. Dis..

[53] Antrobus R.D., Lillie P.J., Berthoud T.K., Spencer A.J., McLaren J.E., Ladell K., Lambe T., Milicic A., Price D.A., Hill A.V. (2012). A T cell-inducing influenza vaccine for the elderly: Safety and immunogenicity of MVA-NP+M1 in adults aged over 50 years. PLoS ONE.

[54] Mullarkey C.E., Boyd A., van Laarhoven A., Lefevre E.A., Veronica Carr B., Baratelli M., Molesti E., Temperton N.J., Butter C., Charleston B. (2013). Improved adjuvanting of seasonal influenza vaccines: Preclinical studies of MVA-NP+M1 coadministration with inactivated influenza vaccine. Eur. J. Immunol..

[55] Antrobus R.D., Berthoud T.K., Mullarkey C.E., Hoschler K., Coughlan L., Zambon M., Hill A.V., Gilbert S.C. (2014). Coadministration of seasonal influenza vaccine and MVA-NP+M1 simultaneously achieves potent humoral and cell-mediated responses. Mol. Ther..

[56] Improved Novel VaccIne CombinaTion InflUenza Study. https://ClinicalTrials.gov/show/NCT03300362.

[57] Kreijtz J.H., Goeijenbier M., Moesker F.M., van den Dries L., Goeijenbier S., De Gruyter H.L., Lehmann M.H., Mutsert G., van de Vijver D.A., Volz A. (2014). Safety and immunogenicity of a modified-vaccinia-virus-Ankara-based influenza A H5N1 vaccine: A randomised, double-blind phase 1/2a clinical trial. Lancet Infect. Dis..

[58] De Vries R.D., De Gruyter H.L., Bestebroer T.M., Pronk M., Fouchier R.A., Osterhaus A.D., Sutter G., Kreijtz J.H., Rimmelzwaan G.F. (2015). Induction of influenza (H5N8) antibodies by modified vaccinia virus Ankara H5N1 vaccine. Emerg. Infect. Dis..

[59] Yoshida R., Igarashi M., Ozaki H., Kishida N., Tomabechi D., Kida H., Ito K., Takada A. (2009). Cross-protective potential of a novel monoclonal antibody directed against antigenic site B of the hemagglutinin of influenza A viruses. PLoS Pathog..

[60] Lee P.S., Yoshida R., Ekiert D.C., Sakai N., Suzuki Y., Takada A., Wilson I.A. (2012). Heterosubtypic antibody recognition of the influenza virus hemagglutinin receptor binding site enhanced by avidity. Proc. Natl. Acad. Sci. USA.

[61] Ekiert D.C., Kashyap A.K., Steel J., Rubrum A., Bhabha G., Khayat R., Lee J.H., Dillon M.A., O’Neil R.E., Faynboym A.M. (2012). Cross-neutralization of influenza A viruses mediated by a single antibody loop. Nature.

[62] Whittle J.R., Zhang R., Khurana S., King L.R., Manischewitz J., Golding H., Dormitzer P.R., Haynes B.F., Walter E.B., Moody M.A. (2011). Broadly neutralizing human antibody that recognizes the receptor-binding pocket of influenza virus hemagglutinin. Proc. Natl. Acad. Sci. USA.

[63] Schmidt A.G., Therkelsen M.D., Stewart S., Kepler T.B., Liao H.X., Moody M.A., Haynes B.F., Harrison S.C. (2015). Viral receptor-binding site antibodies with diverse germline origins. Cell.

[64] Schmidt A.G., Xu H., Khan A.R., O’Donnell T., Khurana S., King L.R., Manischewitz J., Golding H., Suphaphiphat P., Carfi A. (2013). Preconfiguration of the antigen-binding site during affinity maturation of a broadly neutralizing influenza virus antibody. Proc. Natl. Acad. Sci. USA.

[65] Chen C., Liu L., Xiao Y., Cui S., Wang J., Jin Q. (2018). Structural Insight into a Human Neutralizing Antibody against Influenza Virus H7N9. J. Virol..

[66] Hancock K., Veguilla V., Lu X., Zhong W., Butler E.N., Sun H., Liu F., Dong L., DeVos J.R., Gargiullo P.M. (2009). Cross-reactive antibody responses to the 2009 pandemic H1N1 influenza virus. N. Engl. J. Med..

[67] Wrammert J., Koutsonanos D., Li G.M., Edupuganti S., Sui J., Morrissey M., McCausland M., Skountzou I., Hornig M., Lipkin W.I. (2011). Broadly cross-reactive antibodies dominate the human B cell response against 2009 pandemic H1N1 influenza virus infection. J. Exp. Med..

[68] Margine I., Hai R., Albrecht R.A., Obermoser G., Harrod A.C., Banchereau J., Palucka K., Garcia-Sastre A., Palese P., Treanor J.J. (2013). H3N2 influenza virus infection induces broadly reactive hemagglutinin stalk antibodies in humans and mice. J. Virol..

[69] Miller M.S., Tsibane T., Krammer F., Hai R., Rahmat S., Basler C.F., Palese P. (2013). 1976 and 2009 H1N1 influenza virus vaccines boost anti-hemagglutinin stalk antibodies in humans. J. Infect. Dis..

[70] Thomson C.A., Wang Y., Jackson L.M., Olson M., Wang W., Liavonchanka A., Keleta L., Silva V., Diederich S., Jones R.B. (2012). Pandemic H1N1 Influenza Infection and Vaccination in Humans Induces Cross-Protective Antibodies that Target the Hemagglutinin Stem. Front. Immunol..

[71] Sui J., Sheehan J., Hwang W.C., Bankston L.A., Burchett S.K., Huang C.Y., Liddington R.C., Beigel J.H., Marasco W.A. (2011). Wide prevalence of heterosubtypic broadly neutralizing human anti-influenza A antibodies. Clin. Infect. Dis..

[72] Liu L., Nachbagauer R., Zhu L., Huang Y., Xie X., Jin S., Zhang A., Wan Y., Hirsh A., Tian D. (2017). Induction of Broadly Cross-Reactive Stalk-Specific Antibody Responses to Influenza Group 1 and Group 2 Hemagglutinins by Natural H7N9 Virus Infection in Humans. J. Infect. Dis..

[73] He W., Mullarkey C.E., Duty J.A., Moran T.M., Palese P., Miller M.S. (2015). Broadly neutralizing anti-influenza virus antibodies: Enhancement of neutralizing potency in polyclonal mixtures and IgA backbones. J. Virol..

[74] Henry C., Palm A.E., Krammer F., Wilson P.C. (2018). From Original Antigenic Sin to the Universal Influenza Virus Vaccine. Trends Immunol..

[75] Krammer F. (2017). Strategies to induce broadly protective antibody responses to viral glycoproteins. Expert Rev. Vaccines.

[76] Neu K.E., Henry Dunand C.J., Wilson P.C. (2016). Heads, stalks and everything else: How can antibodies eradicate influenza as a human disease?. Curr. Opin. Immunol..

[77] Safety and Immunogenicity of a Live-Attenuated Universal Flu Vaccine Followed by an Inactivated Universal Flu Vaccine. https://ClinicalTrials.gov/show/NCT03300050.

[78] Yassine H.M., Boyington J.C., McTamney P.M., Wei C.J., Kanekiyo M., Kong W.P., Gallagher J.R., Wang L., Zhang Y., Joyce M.G. (2015). Hemagglutinin-stem nanoparticles generate heterosubtypic influenza protection. Nat. Med..

[79] Impagliazzo A., Milder F., Kuipers H., Wagner M.V., Zhu X., Hoffman R.M., van Meersbergen R., Huizingh J., Wanningen P., Verspuij J. (2015). A stable trimeric influenza hemagglutinin stem as a broadly protective immunogen. Science.

[80] Kanekiyo M., Wei C.J., Yassine H.M., McTamney P.M., Boyington J.C., Whittle J.R., Rao S.S., Kong W.P., Wang L., Nabel G.J. (2013). Self-assembling influenza nanoparticle vaccines elicit broadly neutralizing H1N1 antibodies. Nature.

[81] Van de Sandt C.E., Hillaire M.L., Geelhoed-Mieras M.M., Osterhaus A.D., Fouchier R.A., Rimmelzwaan G.F. (2015). Human Influenza A Virus-Specific CD8+ T-Cell Response Is Long-lived. J. Infect. Dis..

[82] Angeletti D., Yewdell J.W. (2017). Is It Possible to Develop a “Universal” Influenza Virus Vaccine? Outflanking Antibody Immunodominance on the Road to Universal Influenza Vaccination. Cold Spring Harb. Perspect. Biol..

[83] Castilla J., Martinez-Baz I., Martinez-Artola V., Reina G., Pozo F., Garcia Cenoz M., Guevara M., Moran J., Irisarri F., Arriazu M. (2013). Decline in influenza vaccine effectiveness with time after vaccination, Navarre, Spain, season 2011/12. Euro Surveill..

[84] Kissling E., Nunes B., Robertson C., Valenciano M., Reuss A., Larrauri A., Cohen J.M., Oroszi B., Rizzo C., Machado A. (2016). I-MOVE multicentre case-control study 2010/11 to 2014/15: Is there within-season waning of influenza type/subtype vaccine effectiveness with increasing time since vaccination?. Euro Surveill..

[85] Wakim L.M., Smith J., Caminschi I., Lahoud M.H., Villadangos J.A. (2015). Antibody-targeted vaccination to lung dendritic cells generates tissue-resident memory CD8 T cells that are highly protective against influenza virus infection. Mucosal. Immunol..

[86] Pichyangkul S., Yongvanitchit K., Limsalakpetch A., Kum-Arb U., Im-Erbsin R., Boonnak K., Thitithayanont A., Jongkaewwattana A., Wiboon-ut S., Mongkolsirichaikul D. (2015). Tissue Distribution of Memory T and B Cells in Rhesus Monkeys following Influenza A Infection. J. Immunol..

[87] Zens K.D., Chen J.K., Farber D.L. (2016). Vaccine-generated lung tissue-resident memory T cells provide heterosubtypic protection to influenza infection. JCI Insight.

[88] Pizzolla A., Nguyen T.H., Sant S., Jaffar J., Loudovaris T., Mannering S.I., Thomas P.G., Westall G.P., Kedzierska K., Wakim L.M. (2018). Influenza-specific lung-resident memory T cells are proliferative and polyfunctional and maintain diverse TCR profiles. J. Clin. Investig..

[89] Kumar B.V., Ma W., Miron M., Granot T., Guyer R.S., Carpenter D.J., Senda T., Sun X., Ho S.H., Lerner H. (2017). Human Tissue-Resident Memory T Cells Are Defined by Core Transcriptional and Functional Signatures in Lymphoid and Mucosal Sites. Cell Rep..

[90] Kumar B.V., Connors T.J., Farber D.L. (2018). Human T Cell Development, Localization, and Function throughout Life. Immunity.

[91] Braciale T.J. (1977). Immunologic recognition of influenza virus-infected cells. II. Expression of influenza A matrix protein on the infected cell surface and its role in recognition by cross-reactive cytotoxic T cells. J. Exp. Med..

[92] Chen Y.Q., Wohlbold T.J., Zheng N.Y., Huang M., Huang Y., Neu K.E., Lee J., Wan H., Rojas K.T., Kirkpatrick E. (2018). Influenza Infection in Humans Induces Broadly Cross-Reactive and Protective Neuraminidase-Reactive Antibodies. Cell.

[93] Grant E.J., Quinones-Parra S.M., Clemens E.B., Kedzierska K. (2016). Corrigendum to ‘Human influenza viruses and CD8+ T cell responses’. Curr. Opin. Virol..

[94] Gras S., Kedzierski L., Valkenburg S.A., Laurie K., Liu Y.C., Denholm J.T., Richards M.J., Rimmelzwaan G.F., Kelso A., Doherty P.C. (2010). Cross-reactive CD8+ T-cell immunity between the pandemic H1N1-2009 and H1N1-1918 influenza A viruses. Proc. Natl. Acad. Sci. USA.

[95] Wu C., Zanker D., Valkenburg S., Tan B., Kedzierska K., Zou Q.M., Doherty P.C., Chen W. (2011). Systematic identification of immunodominant CD8+ T-cell responses to influenza A virus in HLA-A2 individuals. Proc. Natl. Acad. Sci. USA.

[96] Ahmed S.S., Volkmuth W., Duca J., Corti L., Pallaoro M., Pezzicoli A., Karle A., Rigat F., Rappuoli R., Narasimhan V. (2015). Antibodies to influenza nucleoprotein cross-react with human hypocretin receptor 2. Sci. Transl. Med..

[97] A Follow-On Study with an H5 Influenza Vaccine for Subjects Who Participated in Study FLU-001. https://ClinicalTrials.gov/show/NCT01403155.

[98] Safety Study of Recombinant M2e Influenza-A Vaccine in Healthy Adults. https://ClinicalTrials.gov/show/NCT00819013.

[99] Comparative Safety and Immunogenicity of 1.0 µg Intramuscular (i.m.) and 2.0 µg Subcutaneous (s.c.) Dosing with VAX102 (M2e-flagellin) Universal Influenza Vaccine in Healthy Adults. https://ClinicalTrials.gov/show/NCT00921947.

[100] Pleguezuelos O., Robinson S., Fernandez A., Stoloff G.A., Mann A., Gilbert A., Balaratnam G., Wilkinson T., Lambkin-Williams R., Oxford J. (2015). A Synthetic Influenza Virus Vaccine Induces a Cellular Immune Response That Correlates with Reduction in Symptomatology and Virus Shedding in a Randomized Phase Ib Live-Virus Challenge in Humans. Clin. Vaccine Immunol..

[101] Van Doorn E., Liu H., Ben-Yedidia T., Hassin S., Visontai I., Norley S., Frijlink H.W., Hak E. (2017). Evaluating the immunogenicity and safety of a BiondVax-developed universal influenza vaccine (Multimeric-001) either as a standalone vaccine or as a primer to H5N1 influenza vaccine: Phase IIb study protocol. Medicine.

[102] Van Doorn E., Pleguezuelos O., Liu H., Fernandez A., Bannister R., Stoloff G., Oftung F., Norley S., Huckriede A., Frijlink H.W. (2017). Evaluation of the immunogenicity and safety of different doses and formulations of a broad spectrum influenza vaccine (FLU-v) developed by SEEK: Study protocol for a single-center, randomized, double-blind and placebo-controlled clinical phase IIb trial. BMC Infect. Dis..

